# Studies on the mechanism of *Toxoplasma gondii* Chinese 1 genotype Wh6 strain causing mice abnormal cognitive behavior

**DOI:** 10.1186/s13071-022-05618-8

**Published:** 2023-01-25

**Authors:** Qing Tao, Di Yang, Kunpeng Qin, Lei Liu, Mengmeng Jin, Famin Zhang, Jinjin Zhu, Jie Wang, Qingli Luo, Jian Du, Li Yu, Jilong Shen, Deyong Chu

**Affiliations:** 1 Department of Pathogen Biology, Anhui Province Key Laboratory of Microbiology & Parasitology, Anhui Provincial Laboratory of Zoonoses of High Institutions, School of Basic Medicine, Anhui Medical University, Hefei, China; 2 Department of Orthopaedics, the First Affiliated Hospital of Anhui Medical University, Anhui, China; 3 Department of Blood Transfusion, Division of Life Sciences and Medicine, The First Affiliated Hospital of USTC, University of Science and Technology of China, Hefei, China; 4 Maternity and Child Health Hospital of Anhui Province, The Affiliated Maternity and Child Health Hospital of Anhui Medical University, Hefei, China; 5 Department of Biochemistry and Molecular Biology, School of Basic Medical Sciences, Anhui Medical University, Hefei, China; 6 Department of Microbiology and Parasitology, Anhui Provincial Laboratory of Microbiology and Parasitology, Anhui Provincial Laboratory of Zoonoses of High Institutions, School of Basic Medical Sciences, Anhui Medical University, Hefei, China

**Keywords:** *Toxoplasma gondii* Chinese 1 genotype Wh6 strain, Cognitive behavior, Hippocampal neuron, Apoptosis, Aβ, Inflammatory response

## Abstract

**Background:**

Alzheimer's disease presents an abnormal cognitive behavior. TgCtwh6 is one of the predominant *T. gondii* strains prevalent in China. Although *T. gondii* type II strain infection can cause host cognitive behavioral abnormalities, we do not know whether TgCtwh6 could also cause host cognitive behavioral changes. So, in this study, we will focus on the effect of TgCtwh6 on mouse cognitive behavior and try in vivo and in vitro to explore the underlying mechanism by which TgCtwh6 give rise to mice cognitive behavior changes at the cellular and molecular level.

**Methods:**

C57BL/6 mice were infected orally with TgCtwh6 cysts. From day 90 post-infection on, all mice were conducted through the open field test and then Morris water maze test to evaluate cognitive behavior. The morphology and number of cells in hippocampus were examined with hematoxylin-eosin (H&E) and Nissl staining; moreover, Aβ protein in hippocampus was determined with immunohistochemistry and thioflavin S plaque staining. Synaptotagmin 1, apoptosis-related proteins, BACE1 and APP proteins and genes from hippocampus were assessed by western blotting or qRT-PCR. Hippocampal neuronal cell line or mouse microglial cell line was challenged with TgCtwh6 tachyzoites and then separately cultured in a well or co-cultured in a transwell device. The target proteins and genes were analyzed by immunofluorescence staining, western blotting and qRT-PCR. In addition, mouse microglial cell line polarization state and hippocampal neuronal cell line apoptosis were estimated using flow cytometry assay.

**Results:**

The OFT and MWMT indicated that infected mice had cognitive behavioral impairments. The hippocampal tissue assay showed abnormal neuron morphology and a decreased number in infected mice. Moreover, pro-apoptotic proteins, as well as BACE1, APP and Aβ proteins, increased in the infected mouse hippocampus. The experiments in vitro showed that pro-apoptotic proteins and *p*-NF-κBp65, NF-κBp65, BACE1, APP and Aβ proteins or genes were significantly increased in the infected HT22. In addition, CD80, pro-inflammatory factors, notch, hes1 proteins and genes were enhanced in the infected BV2. Interestingly, not only the APP and pro-apoptotic proteins in HT22, but also the apoptosis rate of HT22 increased after the infected BV2 were co-cultured with the HT22 in a transwell device.

**Conclusions:**

Neuron apoptosis, Aβ deposition and neuroinflammatory response involved with microglia polarization are the molecular and cellular mechanisms by which TgCtwh6 causes mouse cognitive behavioral abnormalities.

**Graphical Abstract:**

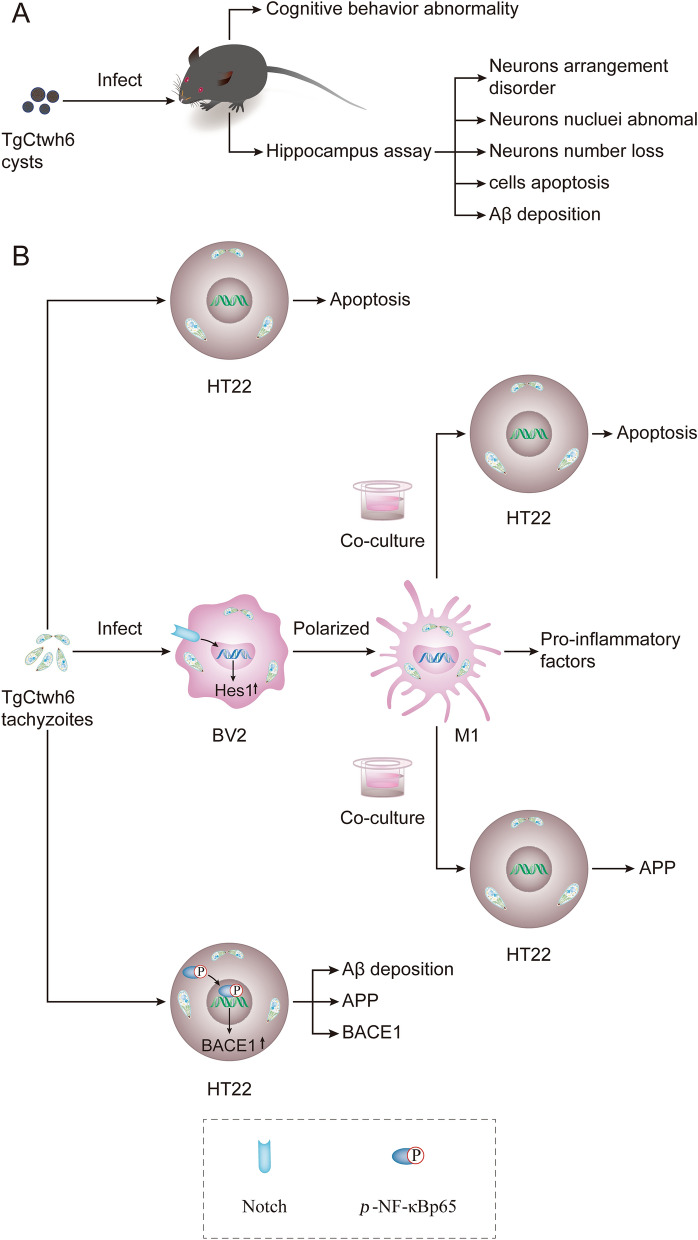

## Background

Alzheimer's disease (AD) presents an abnormal cognitive behavior with a slow progression. Although AD affects millions of people every day, its etiology and pathogenesis remain elusive. AD is characterized by two fundamental pathologies, the presence of beta-amyloid (Aβ) plaques and neurofibrillary tangles [1]. Parasites have been shown to be involved in AD pathogenesis [2, 3].

The neurotropic parasite *Toxoplasma gondii* (*T. gondii*) can invade almost all warm-blooded animals, including humans. Chronic *T. gondii* infection affects almost one-third of the global population [4, 5]. Numerous epidemiological statistics suggested that the positive rate of serum *Toxoplasma* antibodies in AD patients was higher than that in the control group [6]. *Toxoplasma gondii* can enter the brain from various anatomical sites to form tissue cysts through the blood-brain barrier and erode neurons, astrocytes and microglia [7–9]. Increasing evidence indicates that chronic *T. gondii* infection can cause cognitive behavioral abnormalities [10, 11]. Other research elucidates that *T. gondii* infection can induce or aggravate human cognitive impairment, memory loss and learning decline [12]. Cognitive behavioral abnormalities principally include changes in memory, comprehension, learning and judgment and are accompanied by abnormalities in emotions and behaviors [13]. *Toxoplasma gondii* ME49 strain can induce two major hallmarks of AD to produce Aβ protein and hyper-phosphorylated tau protein [14, 15]. Aβ derives from amyloid precursor protein (APP) through proteolytic cleavage by β-secretase 1 (BACE1). It has been reported that in neurological diseases, NF-κBp65 can increase Aβ production by upregulating the activity of the BACE1 protein [16, 17]. Neuronal loss and synapse density decline were also found in the brain of mice infected with *T. gondii* Pru strain, which could cause cognitive behavioral abnormalities [18]. The inflammation response of microglia is also implicated in the development of AD [19]. BV2, a mouse microglial cell line, exhibits a classically activated macrophage (M1) status and secretes proinflammatory factors after stimulation by LPS, IFN-γ or parasites [20, 21]. Furthermore, the Notch signaling pathway is closely related to the activation of microglia and the secretion of proinflammatory mediators from microglia [22].

The predominant genotype of *T. gondii* prevalent in China is Chinese1 (ToxoDB #9) found by our research group. The genotype has two representative strains, *T. gondii* Chinese 1 genotype Wh3 strain (TgCtwh3) and *T. gondii* Chinese 1 genotype Wh6 strain (TgCtwh6). However, to our knowledge no study has investigated the relationship between TgCtwh6 infection and host cognitive behavior changes so far. Therefore, in this study, we will focus on the effect of TgCtwh6 on mouse cognitive behavior and try in vivo and in vitro to explore the mechanism through which TgCtwh6 causes mouse cognitive behavior abnormalities at the cellular and molecular level.

## Methods

### Materials

Hematoxylin and eosin (H&E), Nissl staining kit, thioflavin S, ammonium pyrrolidinedithiocarbamate (PDTC, an inhibitor of NF-κB pathway), 3, 3′-diaminobenzidine tetrahydrochloride (DAB), penicillin and streptomycin were purchased from Sigma (St. Louis, MO, USA). Dulbecco’s modified Eagle’s medium (DMEM) and fetal bovine serum (FBS) were obtained from Wisent (Montreal, QC, Canada). BCA protein assay kit, TRIzol reagent, 2-(4-amidinophenyl)-6-indolecarbamidine dihydrochloride (DAPI) and SDS polyacrylamide gel electrophoresis were purchased from Beyotime (Shanghai, China). RIPA lysis buffer and nitrocellulose membrane were provided by Millipore (Billerica, MA, USA). Anti-NF-κBp65, *p*-NF-κBp65, BACE1, APP, caspase 3, Bax, Bcl-XL, Notch, Hes1 and GAPDH antibodies were purchased from Cell Signaling Technology (Beverly, MA, USA). Anti-Aβ antibody was purchased from Abcam (Cambridge, MA, USA). Anti-synaptotagmin 1 and horseradish peroxide (HRP)-conjugated goat anti-rabbit IgG were purchased from Proteintech (Chicago, IL, USA). Alexa Fluor 488-conjugated anti-rabbit antibody was purchased from Invitrogen (Carlsbad, CA, USA). Prime Script First Strand cDNA Synthesis Kit and SYBR Premix Ex Taq kit were purchased from TaKaRa (TaKaRa Bio, Japan). FITC-labeled anti-mouse CD80, APC-labeled anti-mouse CD206 and Annexin V-FITC Apoptosis Detection Kit I were obtained from BD Biosciences (New York, BD, USA) for flow cytometry (FCM) analysis. The tachyzoites and cysts of TgCtwh6 were maintained in human foreskin fibroblast line (HFF) and mice, respectively, in our laboratory. Hippocampal neuronal cell line (HT22) was purchased from Procell (Wuhan, China). HFF and mouse microglial cell line (BV2) were obtained from Chinese Academy of Sciences (Shanghai, China).

### Animals

Forty-eight male C57BL/6 mice (8- to 12-week-old, specific pathogen free, SPF) weighing from 20 to 22 g were purchased from the Changzhou Cavens Laboratory Animal Company, China (production permit number: Scxk 2016–0010). All animal work was conducted in strict accordance with the Chinese National Institute of Health Guide for the Care and Use of Laboratory Animals, and we obtained the permission of the Institutional Review Board of the Institute of Biomedicine at Anhui Medical University (permit no. AMU26093628). The mice were housed in a colony room of Anhui Province Key Laboratory of Microbiology and Parasitology under controlled conditions (12/12 h light/dark cycle and 22 ± 2 °C, humidity 45 ± 5%) with access to standard food and pure water ad libitum.

### *Toxoplasma gondii* strain

TgCtwh6 (Chinese1) tachyzoites were stored at −80 °C in our laboratory (Anhui Province Key Laboratory of Microbiology and Parasitology). Tachyzoites were cultured in HFF cells in Dulbecco's modified Eagle's medium (DMEM) at 37 °C with 5% CO_2_, supplemented with 10% fetal bovine serum and 1% penicillin/streptomycin. Tachyzoites were intraperitoneally injected into C57BL/6 mice to cause chronic infection. After 6 weeks of infection, the brains of chronically infected mice were collected and mechanically homogenized in saline after the mice had been killed. Number of cysts in the homogenate was counted under a light microscope. Then, these cysts were used to challenge mice for our experiments.

### Experimental design

All mice were randomly divided into two groups of 24 each: the control group and the infected group. Each mouse in the infection group was infected orally with 30 TgCtwh6 cysts from brain homogenate diluted in 0.3 ml normal saline; meanwhile, each mouse in the control group received 0.3 ml normal saline orally. On the 90th day post-infection, all mice undersebt the open field test (OFT) and Morris water maze test (MWMT) to evaluate mouse cognitive behavior. OFT was carried out at day 90 followed by MWMT at day 91 to 96 post-infection. Then, all mice were killed on the 97th day post-infection, and mouse brain tissues were collected for histopathological analysis, protein and gene expression detection. HT22 or BV2 was challenged with TgCtwh6 tachyzoites and cultured in a separate well or transwell device; the target proteins and genes were evaluated by immunofluorescence staining, western blotting, qRT-PCR and flow cytometry assay.

### Open field test

Open field test (OFT) was carried out on the open field apparatus with a black inner wall; the apparatus bottom is white with black interwoven lines (Shanghai Bio will Co., Ltd., Shanghai, China) [23, 24]. Briefly, the motor and exploration ability of mice was detected in an open field under the same time and light conditions on the 90th day post-TgCtwh6 cyst infection. Each mouse was placed in the center of a square open field arena (18 × 18 × 18 inches). The movement of the mouse was recorded by a computer-operated tracking system with PC-based video capture software (Mobiledatum Information Technology Co., Ltd., Shanghai, China). The recorded video file was analyzed to determine the average velocity, total distance traveled and time spent in the center and on the periphery (or in the corners) by each mouse during the 5-min observation period.

### Morris Water Maze Test

The MWM apparatus contained a black circular swimming pool, which was divided into four quadrants of equal size. A square platform was hidden 2 cm below the water surface in the center of the southwest quadrant of the pool (target quadrant). The tests were implemented under a dimly light with various geometrical images at different places on the walls around the pool. The performances of the mice were recorded by a computer-operated tracking system with PC-based video capture software (Mobiledatum Information Technology Co., Ltd., Shanghai, China) [25, 26].

Spatial learning (spatial acquisition phase): The spatial acquisition trials were performed for 5 consecutive days. Each mouse was given four trials per day to find the hidden platform. The interval between trials was at least 30 s [25–28]. The mice were individually released from a randomly selected quadrant with their faces toward the wall for each trial in which the direct path to the hidden platform was different each time. Each mouse was given at most 60 s to find the hidden platform. After finding the hidden platform, the mouse was allowed to stay on the platform for 20 s; then, it was removed from the pool and put into its cage for a rest. After 30 s, it was once more put in the pool for the next trial. If the mouse failed to find the platform within given time, a technician placed it on the platform for 15 s. The parameters of escape latency (the time spent in reaching the platform), swimming speed and total traveled distance to reaching the platform were recorded in each trial [26–29].

Spatial memory (probe phase): On the 6th day of the MWMT, the probe trial was conducted to assay spatial memory. The hidden platform was removed from the target quadrant of the pool, and each mouse was released into the water, allowing it to swim freely and search for the previous platform for 60 s. The total time each mouse stayed in the target quadrant (or percentage of total time in the target quadrant) and frequency each mouse entered the target quadrant (or number of platform crossing) were recorded in each trial.

### Histopathological analysis

#### Microscope diagnosis of T. gondii

A piece of tissue was taken from a mouse brain and was mechanically homogenized in saline. The cyst in the homogenate was observed under a light microscope at a magnification of 40 × 100. If a cyst was found in a mouse brain, it suggested that this mouse had been infected by TgCtwh6 successfully.

The right brain was collected and fixed in 4% paraformaldehyde for 24 h at room temperature, and then the brain was dehydrated in alcohol series (from 70 to 100%) followed by embedding in paraffin for histopathological examinations. The remaining brain tissue was stored at −80℃ for RNA and protein assays.

#### H&E and Immunohistochemistry (IHC)

In general, brain tissues embedded in paraffin blocks were cut into 4-μm-thick sections to examine the histopathological changes in the hippocampus tissue by H&E staining. Additionally, in immunohistochemistry assay, after brain tissue sections had been dewaxed in xylene three times, they were rehydrated in gradient concentrations of ethanol and then incubated in sodium citrate solution buffer at 92 °C for 10 min. After that, 0.3% H_2_O_2_ was dropwise added to these sections to deactivate endogenous peroxidase. Finally, these sections were incubated with anti-Aβ (1:200) overnight at 4 °C, followed by incubating with HRP-conjugated goat anti-rabbit secondary antibody for 45 min. Then, the sections were color developed with DAB. All H&E and immuno-stained tissue sections were observed under light microscopy at a magnification of 20 × 100 and 40 × 100, and then the images in five randomly different fields of view of the hippocampal zone for each section were captured by an observer in a blinded manner. Quantitative (optical density of anti-Aβ positive areas in the hippocampus tissue) and qualitative changes were analyzed using morphometric software (Image-Pro Plus software, Media Cybernetics, Inc., Rockville, MD, USA).

#### Nissl staining

Nissl staining was performed to detect changes in the number of neurons after TgCtwh6 infection. Brain sections were made as the method described previously. The frozen sections were stained with 0.1% cresyl violet for 20 min and then rinsed with PBS, dehydrated in a graded alcohol series, cleared with xylene and finally mounted with neutral gum. All sections were observed under light microscopy at a magnification of 20 × 100 and 40 × 100, and then the images in five randomly different fields of view of hippocampal zone for each section were captured by an observer in a blinded manner. The total number of Nissl staining-positive cells in these images was counted using morphometric software.

#### Thioflavin S plaque staining

First, 0.3% thioflavin S solution was prepared with 50% alcohol. Then, the sections were incubated with 0.3% thioflavin S solution at room temperature for 8 min, followed by counterstaining nuclei with DAPI. The sections were placed in PBS and washed three times for 5 min each. Finally, they were observed under a fluorescence microscope at a magnification of 20 × 100 and 40 × 100. Then, the images in five randomly different fields of view of hippocampal zone for each section were photographed by an observer in a blinded manner. The plaque area in these images was measured using morphometric software [15, 30, 31].

### Immunofluorescence staining

TgCtwh6 tachyzoites were harvested from the continuous cell cultures in HFF. HT22s were seeded in a 12-well plate (one coverslip on each well bottom) and divided into two or four groups. After HT22 had been cultured, PDTC (10 mmol/l, an inhibitor of NF-κB) and TgCtwh6 tachyzoites (1 × 10^6^) were added to the corresponding group at different time points, respectively, and then cultured continually. Finally, the HT22s were washed by PBS and fixed in 4% paraformaldehyde. After cell membranes had been penetrated by 0.5% Triton X-100, these cells were incubated with anti-Aβ, *p*-NF-κBp65, NF-κBp65 or BACE1 antibody, respectively, for 16 h. Then, these cells were incubated with Alexa Fluor 488-conjugated anti-rabbit antibody for 1 h at room temperature, followed by counterstaining nuclei with DAPI [15, 32]. Coverslip was mounted on glass slide and observed under a fluorescence microscope at a magnification of 40 × 100. Then, the images in five randomly different fields of view for each coverslip were photographed by an observer in a blinded manner. The percentage of positively stained cells was calculated using morphometric software.

### Western blotting analysis

Around 100 mg of mouse hippocampus tissue or the cultured HT22 and BV2 cells were lysed in the ice-cold RIPA lysis buffer supplemented with protease inhibitors, and the total protein concentrations were detected using BCA protein assay kit. The proteins (20 µg) from each sample were added to 10% polyacrylamide gels and separated and electrophoretically transferred to a nitrocellulose membrane. Non-specific binding in protein-transferred nitrocellulose membranes was blocked with 5% skim milk in PBS-Tween-20 (0.1%) for 2 h at room temperature. The membranes were incubated with anti-APP (1:1,000), BACE1 (1:1000), NF-κBp65 (1:1,000), *p*-NF-κBp65 (1:1000), Bax (1:500), Bcl-XL (1:500), caspase 3 (1:500), synaptotagmin 1 (1:1000), Hes1 (1:500), Notch (1:500) and GAPDH (1:2,000) antibodies, respectively, at 4 °C overnight, and then with HRP-conjugated secondary antibody for 1 h at room temperature. The specific protein signals were captured by ECL kit. The protein bands in images were visualized by Bio-Rad XRS imaging system, and the protein intensity was semiquantitatively evaluated by an observer in a blinded manner using image J software [14, 33].

### Quantitative Real-Time PCR (RT-qPCR)

RNA of the mouse hippocampus tissue or the cultured HT22 and BV2 cells was extracted using the TRIzol reagent followed by determining RNA concentration and purity by NanoDrop2000 (Thermo Scientific, Shanghai, China). RNA 1 μg was reversely transcribed to cDNA using Prime Script First Strand cDNA Synthesis Kit. The qRT-PCR was performed to detect the expression of BACE1, APP, IL-6, TNF-α, iNOS and TGF-β1 using SYBR Premix Ex Taq kit via Light Cycler 480 (Roche, Switzerland), and the thermal cycling condition was programmed following the manufacturer’s protocol. Cycle threshold values were counted through 2^−ΔΔCT^ method to analyze the relative gene expression. GAPDH, a housekeeping gene, playing the role of internal reference, was used as a control for the relative quantitative evaluation of the transcript abundance of target RNA [34, 35]. Sequences of gene-specific primers are listed in Table 1.

**Table 1:** Primers used for qRT-PCR

Primers	Forward primer (5′–3′)	Reverse primer (5′–3′)
APP	TGAATGTGCAGAATGGAAAGTG	AACTAGGCAACGGTAAGGAATC
BACE1	GCAGACATGGAAGACTGTGGCTAC	GGCAGAGTGGCAACATGAAGAGG
IL-6	CCGGAGAGGAGACTTCACAG	CATTTCCACGATTTCCCAGA
TNF-α	ACGGCATGGATCTCAAAGAC	GTGGGTGAGGAGCACGTAGT
iNOS	CACCTTGGAGTTCACCCAGT	ACCACTCGTACTTGGGATGC
TGF-β1	CTGGATACCAACTACTGCTTCAG	TTGGTTGTAGAGGGCAAGGACCT
GAPDH	CAACTTTGGCATTGTGGAAGG	ACACATTGGGGGTAGGAACAC

### Cell culture and co-culture system

BV2 and HT22 were cultured separately in DMEM medium supplemented with 10% FBS, 2 mM l-glutamine (Gibco, Grand Island, New York, NY, USA), 100 μg/ml streptomycin and 100 U/ml penicillin at 37 °C with 5% CO_2_ [36]. First, BV2s (1 × 10^6^) were seeded into a 12-well plate and cultured for 12 h. Then, TgCtwh6 tachyzoites (1 × 10^6^) were added into the same plate to infect cells for another 24 h followed by evaluating gene expression of IL-6, TNF-α, iNOS and TGF-β1 in BV2 using qRT-PCR, polarization state of BV2 using FCM, protein expression of Hes1 and Notch using western blotting.

The transwell device (Corning, Corning, NY, USA) was used to establish a co-culture system [37]. The pore size of the polycarbonate filter membrane in the upper chamber was 0.4 μm, which allowed small and soluble molecules but not cells to pass through. In the transwell device, BV2s (1 × 10^6^) were cultured in the upper chamber. After 12 h, TgCtwh6 tachyzoites (1 × 10^6^) were added into the upper chamber to infect BV2 for another 24 h, and then culture supernatants were discarded. Next, the infected BV2 was co-cultured in replenished DMEM for another 24 h with the HT22 seeded in the lower layer of the transwell device. HT22 cells in lower chamber were harvested for APP and apoptosis protein analysis by western blotting and FCM.

Moreover, in the experiment of PDTC inhibiting NF-κB signaling, HT22 cells were seeded in a 12-well plate and divided into four groups: control group, PDTC group, infection group and PDTC + infection group. After 12 h, PDTC was added into each well of the PDTC group or PDTC + infection group to pretreat cells for 12 h. TgCtwh6 tachyzoites were added to the infection group and PDTC + infection group, respectively. After culturing for another 24 h, HT22 cells were used for western blotting or immunofluorescence staining.

### FCM assay

BV2 cells with or without TgCtwh6 infection were collected and washed in PBS containing 1% FBS and then adjusted to 1 × 10^6^ cells per 100 µl PBS. The cells were subjected to FITC-labeled anti-mouse CD80 and APC-labeled anti-mouse CD206 for surface antigen staining. All cells were incubated with these antibodies at 4 ℃ for 30 min, protected from light and washed twice in PBS before detection by FCM for polarization state of BV2. HT22 cells co-cultured with BV2 in the transwell device were collected. The apoptosis of these HT22 cells was assessed using FITC Annexin V apoptosis detection kit with CytoFLEX flowcytometry (Beckman Coulter, USA). The results were shown and analyzed by an observer in a blinded manner using CytExpert 2.1 software [10].

### Statistical analyses

All data were presented as mean ± standard error of the mean (SEM) and were statistically analyzed using SPSS version 17 statistical package (SPSS Inc., Chicago, IL, USA). All quantifications and analyses were performed by an observer blinded to the treatment conditions. Data from the experiment of PDTC inhibiting NF-κB signaling were analyzed by one-way ANOVA followed by Bonferroni’s post hoc test for multiple comparisons. The other data were analyzed by two-tailed Student’s *t*-test for comparing the difference between two groups. The differences between the groups were considered statistically significant if a *P*-value was < 0.05.

## Results

### TgCtwh6 infection impairs mouse cognitive behavior

**Fig. 1:**
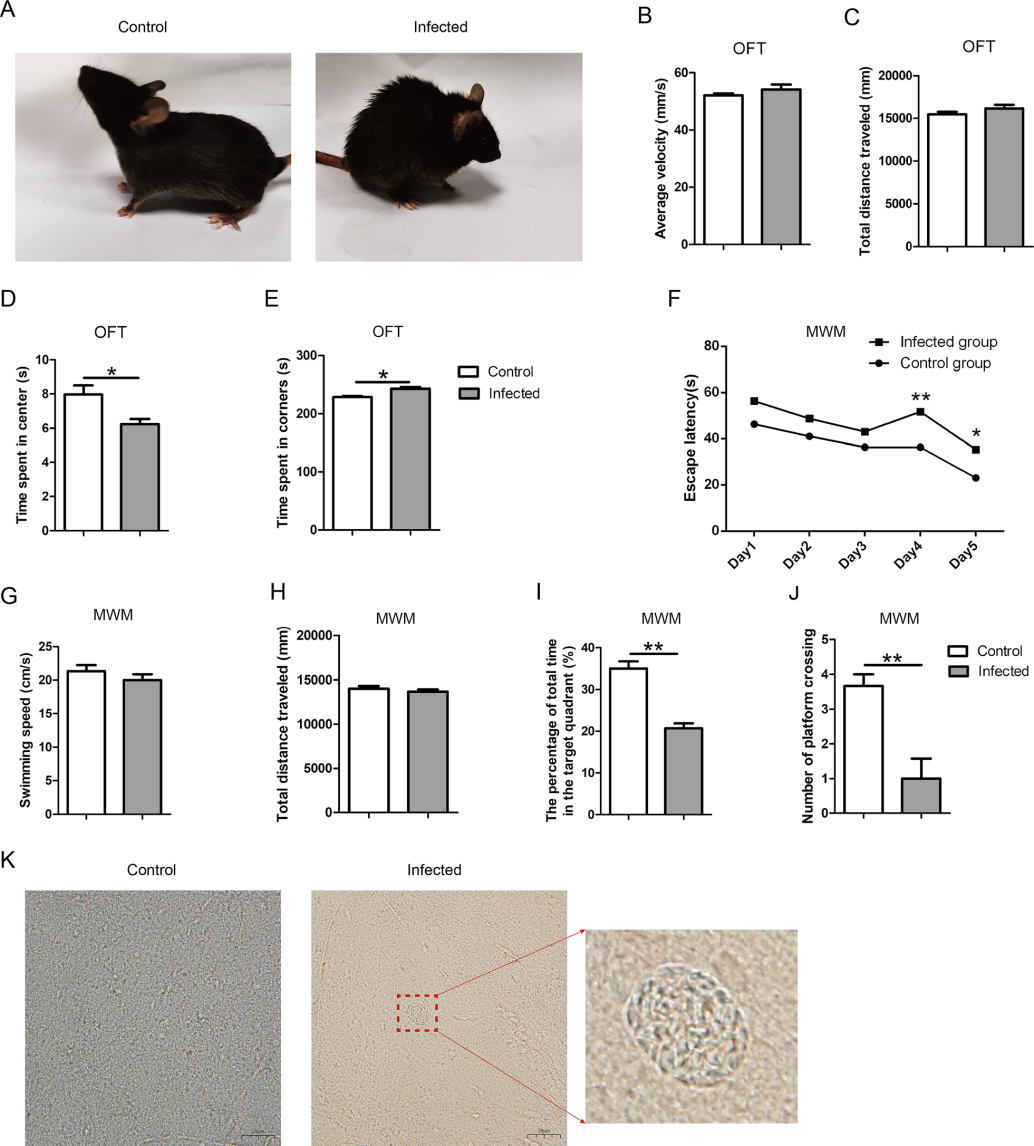
Effects of TgCtwh6 infection on mouse cognitive behavior. Mice were randomly divided into the infected and control groups. Each mouse in the infected group was infected orally with 30 TgCtwh6 cysts. TgCtwh6 infection caused mouse appearance and posture changes (**A**). The motor and exploration abilities were evaluated with OFT on the 90th day post-TgCtwh6 cyst infection (**B**–**E**). The spatial learning ability was measured in MWMT for 5 consecutive days on the 6th day of the MWMT; spatial memory ability was assayed (**F**–**J**). Mice were killed on the 97th day post-infection, and brain cysts were assessed by tissue squash method with light microscopic examination at a magnification of 40 × 100 (Scale bars, 20 μm) (**K**). Data are represented as mean ± SEM and were analyzed by two-tailed Student’s *t*-test for comparing the difference between two groups (*n* = 24 each group). *P < 0. 05, **P < 0.01

To identify whether TgCtwh6 infection can lead to damage of cognitive behavior, we infected the mice with TgCtwh6 cysts. At day 90 post-infection, compared with the non-infected mice, the infected mice usually showed a typical hunchback posture: ruffled piloerection of the fur, stiff limbs, particularly the hind limbs, and whitish eyeballs (Fig. 1A). Then, the OFT was conducted to determine mouse motor and exploration ability. The result showed no significant difference between the two groups at average velocity and total distance traveled by the mouse, which suggested TgCtwh6 infection in mice did not affect their motor ability (data not shown) (Fig. 1B, C); meanwhile, the experiment showed that the infected mice exhibited a preference for the periphery or corners (*t*-test: *t*_(4)_ = 3.986, *P* = 0.0163) and a decline in the range of activity (*t*-test: *t*_(4)_ = 3.181, *P* = 0.0335), which suggested that TgCtwh6 infection made mice anxious and incapable of exploring environment (Fig. 1D, E).

Then, we carried out the MWMT to evaluate spatial learning and memory. The result demonstrated that on the 4th (*t*-test: *t*_(4)_ = 8.573, *P* = 0.001) and 5th (*t*-test: *t*_(4)_ = 44.25, *P* = 0.0132) day of the spatial acquisition trial, the mice in infected group spent more time in identifying and locating the hidden platform than the mice in control, revealing the mouse memory had been impaired by TgCtwh6 infection (Fig. 1F). However, the swimming speed and total distance traveled by mice had no obvious difference between the two groups, indicating again that the motor ability of mice was not impaired (data not shown) (Fig. 1G, H). The infected mice spent less time in the target quadrant (*t*-test: *t*_(4)_ = 7.903, *P* = 0.0014) and had fewer hidden platform crossings (t-test: *t*_(4)_ = 8.578, *P* = 0.001) than the non-infected mice in the probe trial, showing the infected mice spatial learning and memory had been damaged (Fig. 1I, J). In all, these results confirmed that TgCtwh6 can damage mouse cognitive behavior. Mouse brain tissue sections were observed under light microscope, and the results showed that some TgCtwh6 cysts existed in the infected mouse brains (Fig. 1K).

### TgCtwh6 infection causes mouse hippocampal cell apoptosis

**Fig. 2:**
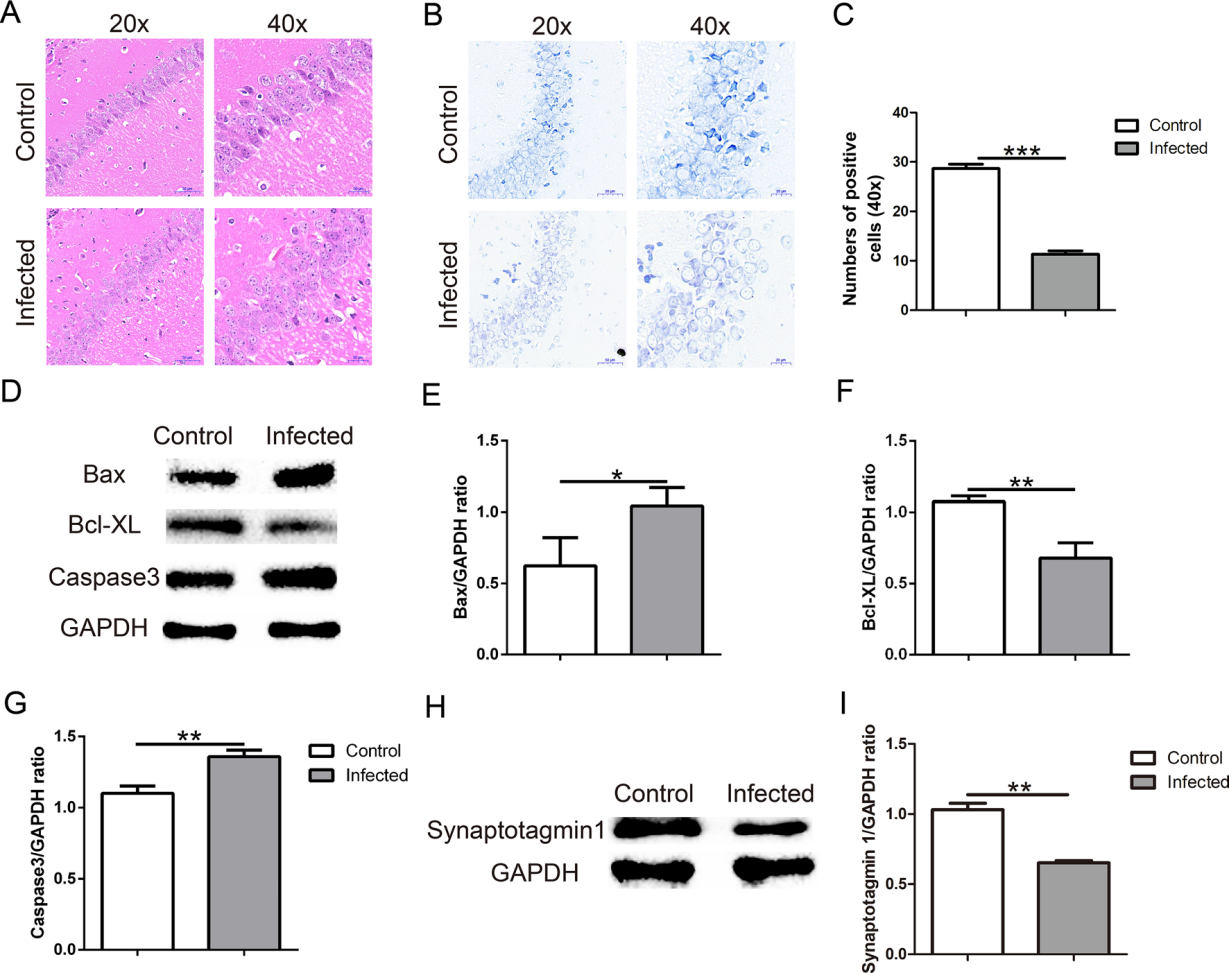
TgCtwh6 infection leads to the apoptosis of hippocampus cells in mouse. The mouse brain tissues were cut into sections for staining. The hippocampus tissue section was first stained with hematoxylin and eosin, and then the neurons were observed under a light microscope at a magnification of 20 × 100 and 40 × 100 (scale bars, 50 μm and 20 μm, respectively) (**A**). Besides, the mouse hippocampus tissue section was stained with Nissl dye, and then the neurons were observed under a light microscope at a magnification of 20 × 100 and 40 × 100 (scale bars, 50 μm and 20 μm, respectively). The number of positive cells in each field of view were evaluated with semiquantitative analysis (**B**, **C**). The mouse right brain hippocampus tissues were lysed and proteins were extracted; then, the apoptosis-related proteins were assessed by western blotting and the protein expression was determined semiquantitatively (**D**–**G**). Synaptotagmin 1 protein from hippocampus tissue was detected by western blotting and analyzed using semiquantitative method (**H**, **I**). Data are represented as mean ± SEM and were analyzed by two-tailed Student’s *t*-test for comparing the difference between the two groups (*n* = 3 each group). *P < 0. 05, **P < 0.01, ***P < 0.001

The hippocampus tissue was evaluated in the following experiments. H&E staining of brain tissue showed that in the normal group the hippocampal zones were uniformly stained with intact tissue and neuron structure; moreover, the nuclear membrane boundaries were clear. On the contrary, in the infected group the hippocampal neurons were disorganized in the arrangement, decreased in the number and stained abnormally deep in nuclei (Fig. 2A). The result indicated that TgCtwh6 infection could cause histopathological changes in the mouse hippocampus. Compared with the control group, the number of Nissl bodies in the hippocampal tissue in the infected group decreased dramatically (Fig. 2B, C), which also indicated that TgCtwh6 infection could lead to a decline in the number of hippocampal neurons (*t*-test: *t*_(4)_ = 11.24, *P* = 0.0004). To determine whether the neuron loss is related to apoptosis, we checked the apoptosis-related proteins in mouse hippocampal tissues by western blotting. The result showed that Bax (*t*-test: *t*_(4)_ = 4.49, *P* = 0.0109) and caspase 3 (*t*-test: *t*_(4)_ = 4.834, *P* = 0.0084) were enhanced, while Bcl-XL (*t*-test: *t*_(4)_ = 6.743, *P* = 0.0025) was diminished, implying TgCtwh6 infection could provoke hippocampal cells, probably including neuron apoptosis (Fig. 2D–G). Furthermore, synaptotagmin 1, a neuron synapse-specific marker, apparently declined in the infected mouse hippocampal tissue (Fig. 2H, I), suggesting that TgCtwh6 infection lowered the number and function of nerve synapses (*t*-test: *t*_(4)_ = 6.971, *P* = 0.0022).

### TgCtwh6 infection induces Aβ immunoreactivity in mouse hippocampus

**Fig. 3:**
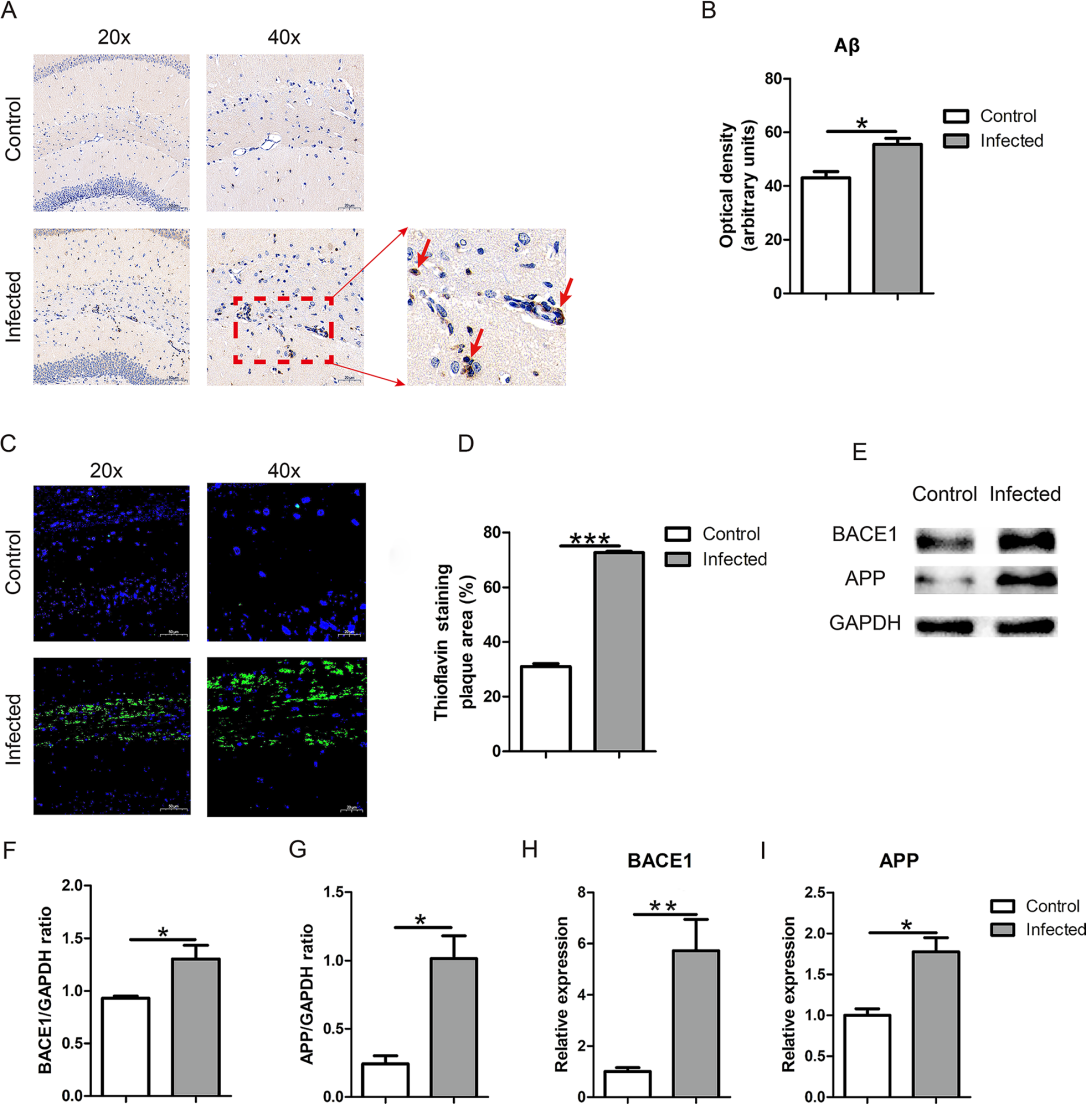
TgCtwh6 infection increases APP, BACE1 and Aβ production in mouse hippocampus tissue. First, the hippocampus tissue section was analyzed by immunhistochemistry method. The stained yellow positive areas were observed under a light microscope at a magnification of 20 × 100 and 40 × 100 (scale bars, 50 μm and 20 μm, respectively) (**A**). The Aβ protein expression in the mouse hippocampal zone was evaluated with semiquantitative method (**B**). Then, the hippocampus tissue section was stained with thioflavin S solution followed by counterstaining nuclei with DAPI. The stained bright green plaques were observed under a fluorescence microscope at a magnification of 20 × 100 and 40 × 100 (scale bars, 50 μm and 20 μm, respectively) (**C**). The Aβ protein expression in the mouse hippocampal zone was estimated using semiquantitative method (**D**). Third, the mouse right brain hippocampus tissues were lysed and proteins were extracted; APP and BACE1 protein were detected by western blotting and then analyzed semiquantitatively (**E**–**G**). Finally, the RNA of the mouse right brain hippocampus tissue was extracted and then reversely transcribed to cDNA. APP and BACE1 gene expressions were assessed by qRT-PCR and then analyzed semiquantitatively (H, I). Data were represented as mean ± SEM and were analyzed by two-tailed Student’s *t*-test for comparing the difference between the two groups (*n* = 3 each group). *P < 0. 05, **P < 0.01, ***P < 0.001

The deposition of Aβ in brain tissue is one of the most important pathological features in Alzheimer's patients. To verify whether TgCtwh6 infection would cause the deposition of Aβ in infected mouse brain, we first compared the production of Aβ in mice of the two groups by IHC and thioflavin S plaque staining. The results of IHC staining showed that Aβ protein expression in the hippocampus of infected mice was significantly higher than in non-infected mice (*t*-test: *t*_(4)_ = 3.244, *P* = 0.0316) (Fig. 3A, B); moreover, thioflavin S plaque staining, a gold standard for Aβ detection, showed that in contrast to the non-infected mouse, there were many bright green plaques stained with thioflavin S in the hippocampus of the infected mouse (*t*-test: *t*_(4)_ = 14.92, *P* = 0.0001) (Fig. 3C, D), implying that Aβ protein expression significantly increased in the infected mouse brain. Finally, western blotting and qRT-PCR results clarified that the expression levels of BACE1 (*t*-test: *t*_(4)_ = 4, *P* = 0.0161, for protein; *t*-test: *t*_(4)_ = 4.351, *P* = 0.0121, for gene), APP (*t*-test: *t*_(4)_ = 4.351, *P* = 0.0121, for protein; *t*-test: *t*_(4)_ = 4.257, *P* = 0.0131 for gene) proteins and genes in the hippocampus of infected mice were significantly upregulated compared with those of non-infected mice (Fig. 3E-I).

### TgCtwh6 tachyzoites directly induce HT22 apoptosis

**Fig. 4:**
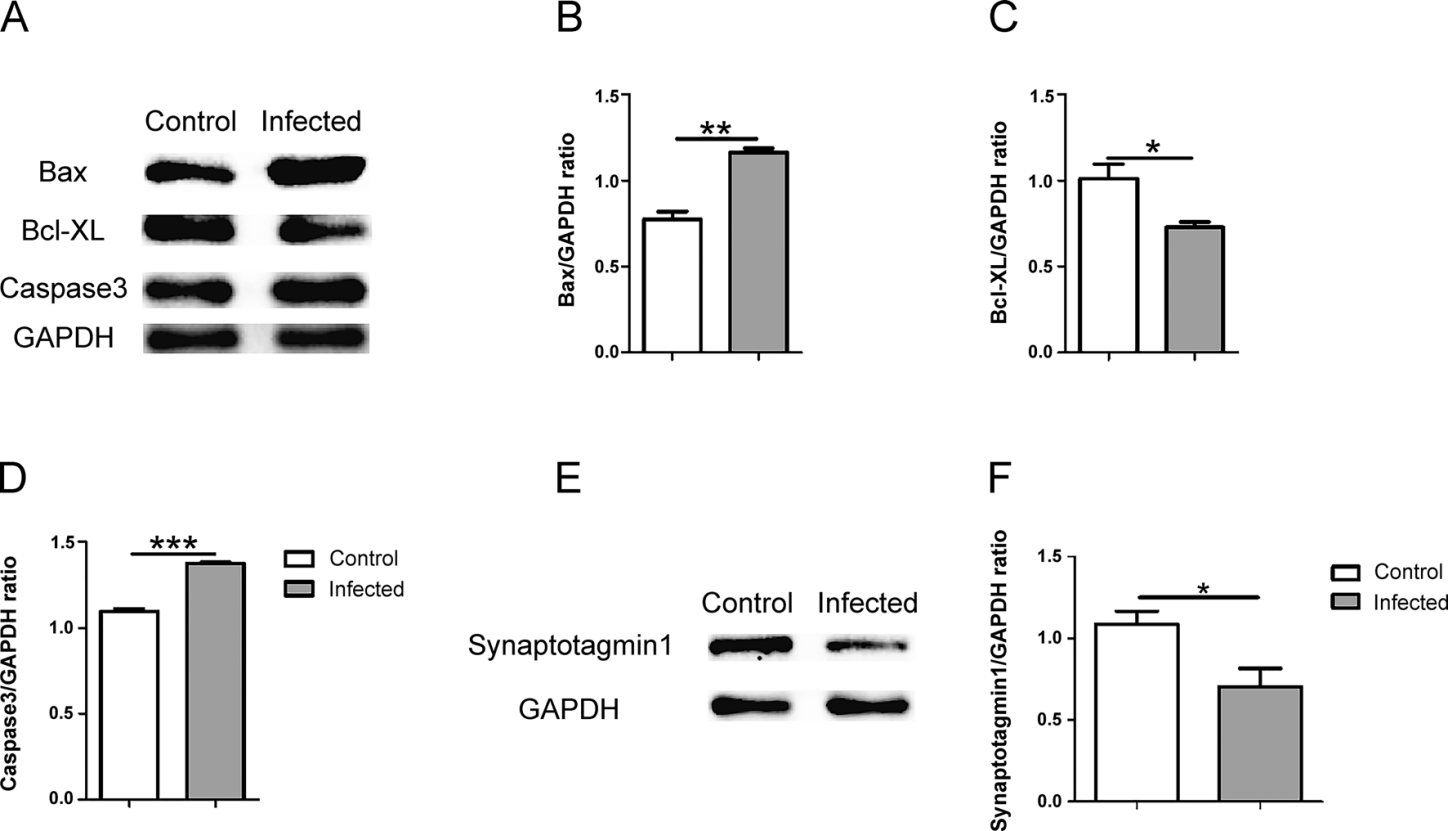
TgCtwh6 tachyzoites affect apoptosis-related and synaptotagmin 1 protein expression in HT22. After TgCtwh6 tachyzoites infected HT22 cells for 24 h, the HT22 cells were lysed and proteins extracted. The expression of apoptosis-related proteins of HT22, such as Bax, Bcl-XL and caspase 3, was measured by western blotting and then analyzed by semiquantitative method (**A**–**D**). Also, synaptotagmin 1 protein in the infected HT22 was detected by western blotting (**E**, **F**). Data were represented as mean ± SEM and were analyzed by two-tailed Student’s *t*-test for comparing the difference between the two groups (*n* = 3 each group). *P < 0. 05, **P < 0.01, ***P < 0.001

Our in vivo experiments showed that TgCtwh6 infection resulted in hippocampal cell apoptosis and hippocampal neuron number loss. To clarify whether TgCtwh6 could give rise to neuron apoptosis, we infected HT22, a mouse hippocampal neuron cell line, with TgCtwh6 tachyzoites to investigate the mechanism with which the infected neuron was lost in vitro. After TgCtwh6 tachyzoites had infected HT22 cells for 24 h, the apoptosis-related proteins of HT22, such as Bax, Bcl-XL and caspase 3, were detected by western blotting. The results showed that the expressions of pro-apoptotic proteins Bax (*t*-test: *t*_(4)_ = 8.139, *P* = 0.0012) and caspase 3 (*t*-test: *t*_(4)_ = 4.602, *P* = 0.01) were significantly increased in the infected HT22 compared with those in the non-infected HT22, while the expression of anti-apoptotic protein Bcl-XL (*t*-test: *t*_(4)_ = 13.19, *P* = 0.0002) was markedly reduced in the infected group compared with the control group (Fig. 4A–D). Besides, compared with the control group, the protein level of synaptotagmin 1 in HT22 also decreased in the infected group (*t*-test: *t*_(4)_ = 4, *P* = 0.0161), suggesting that the number and function of HT22 reduced because of apoptosis induced by TgCtwh6 tachyzoites infection (Fig. 4E, F). Taken together, the results in vivo and in vitro implied that TgCtwh6 infection could possibly promote mouse hippocampal neuron apoptosis, which caused neuron loss and dysfunction.

### BV2 cells infected with TgCtwh6 tachyzoites are polarized to M1 possibly via Notch pathway

**Fig. 5:**
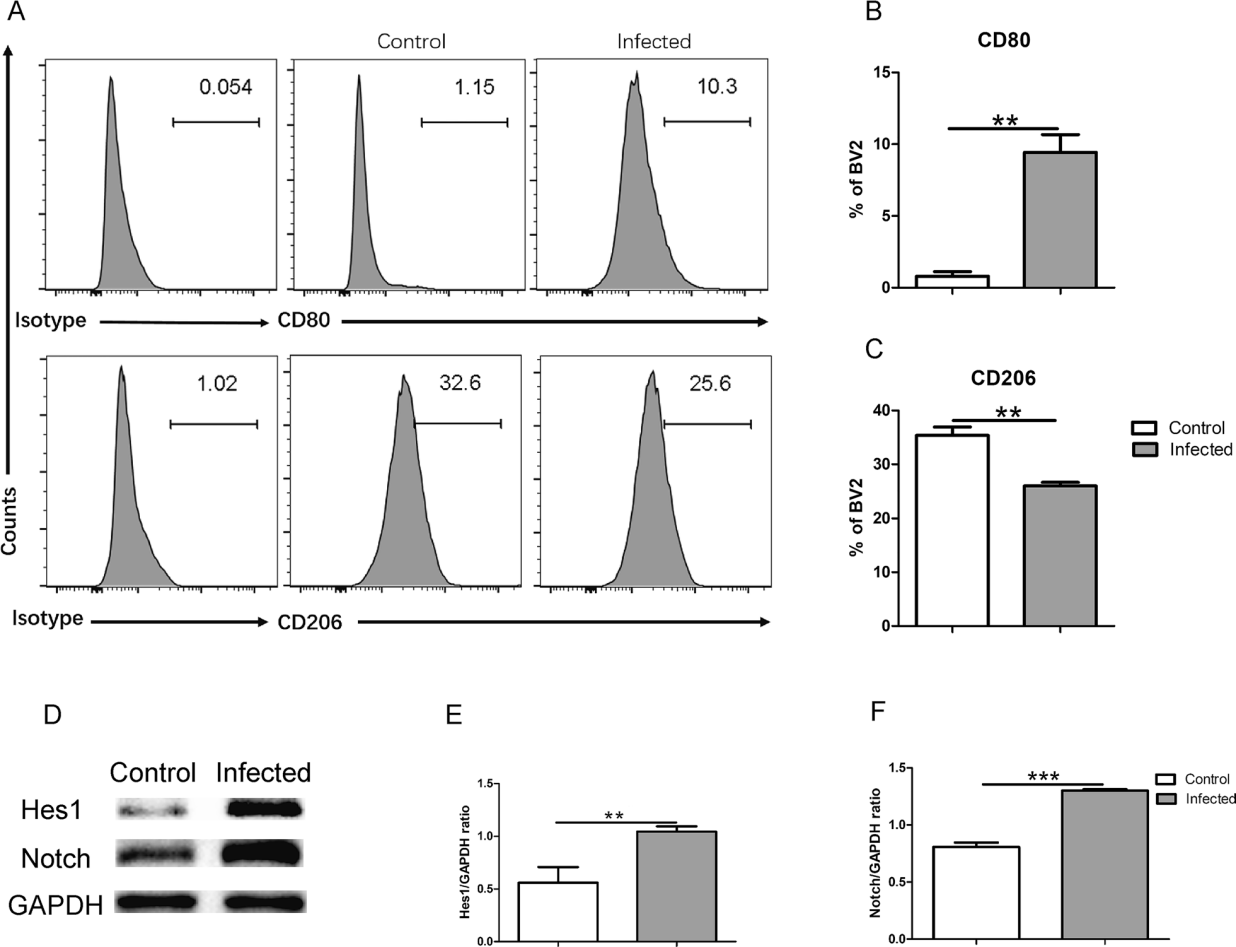
TgCtwh6 tachizoites polarize BV2 to M1 probably via Notch signaling pathway. After BV2 cells were infected by TgCtwh6 tachyzoites for 24 h, BV2 cells were collected and assessed quantitatively for polarization status using FCM (**A**–**C**). Additionally, the infected BV2 cells were lysed and proteins extracted. The expression of Notch and Hes1 proteins was detected by western blotting and then analyzed semiquantitatively (**D**–**F**). Data were represented as mean ± SEM and analyzed by two-tailed Student’s *t*-test for comparing the difference between the two groups (*n* = 3 each group). **P < 0.01, ***P < 0.001

There is some clear evidence that the inflammatory response of microglia is part of the vital pathogenesis of AD [38]. To ascertain the effect of microglia infected by TgCtwh6 on neurons, we first explored in vitro the mechanism with which the TgCtwh6 tachyzoites induce BV2 polarization. In the following experiments, BV2 cells were infected by TgCtwh6 tachyzoites for 24 h; then, BV2 polarization status was determined by FCM according to the CD80 and CD206 expression level. The data indicated that the expression level of CD80 was significantly increased (*t*-test: *t*_(4)_ = 7.658, *P* = 0.0016), while the expression level of CD206 remarkably decreased (*t*-test: *t*_(4)_ = 5.94, *P* = 0.004) (Fig. 5A–C). As is well know, CD80 is a marker of M1 and CD206 is a marker of M2. So, the results suggested that TgCtwh6 tachyzoite infection can cause BV2 to shift towards M1-like phenotype. Subsequently, we analyzed the expression of Notch and Hes1, two key signaling proteins in the Notch pathway during BV2 polarization, to survey the possible mechanism with which BV2 was polarized by TgCtwh6 tachyzoites. The results showed that Notch (*t*-test: *t*_(4)_ = 11.43, *P* = 0.0003) and Hes1 (*t*-test: *t*_(4)_ = 5.696, *P* = 0.0047) protein expressions in TgCtwh6 tachyzoite-infected BV2 significantly increased compared with the non-infected BV2 (Fig. 5D–F). The experiments revealed that TgCtwh6 infection might induce the polarization of BV2 towards M1 by activating Notch signaling.

### BV2 cells infected with TgCtwh6 tachyzoites generate pro-inflammatory factors leading to HT22 apoptosis in co-culture system

**Fig. 6:**
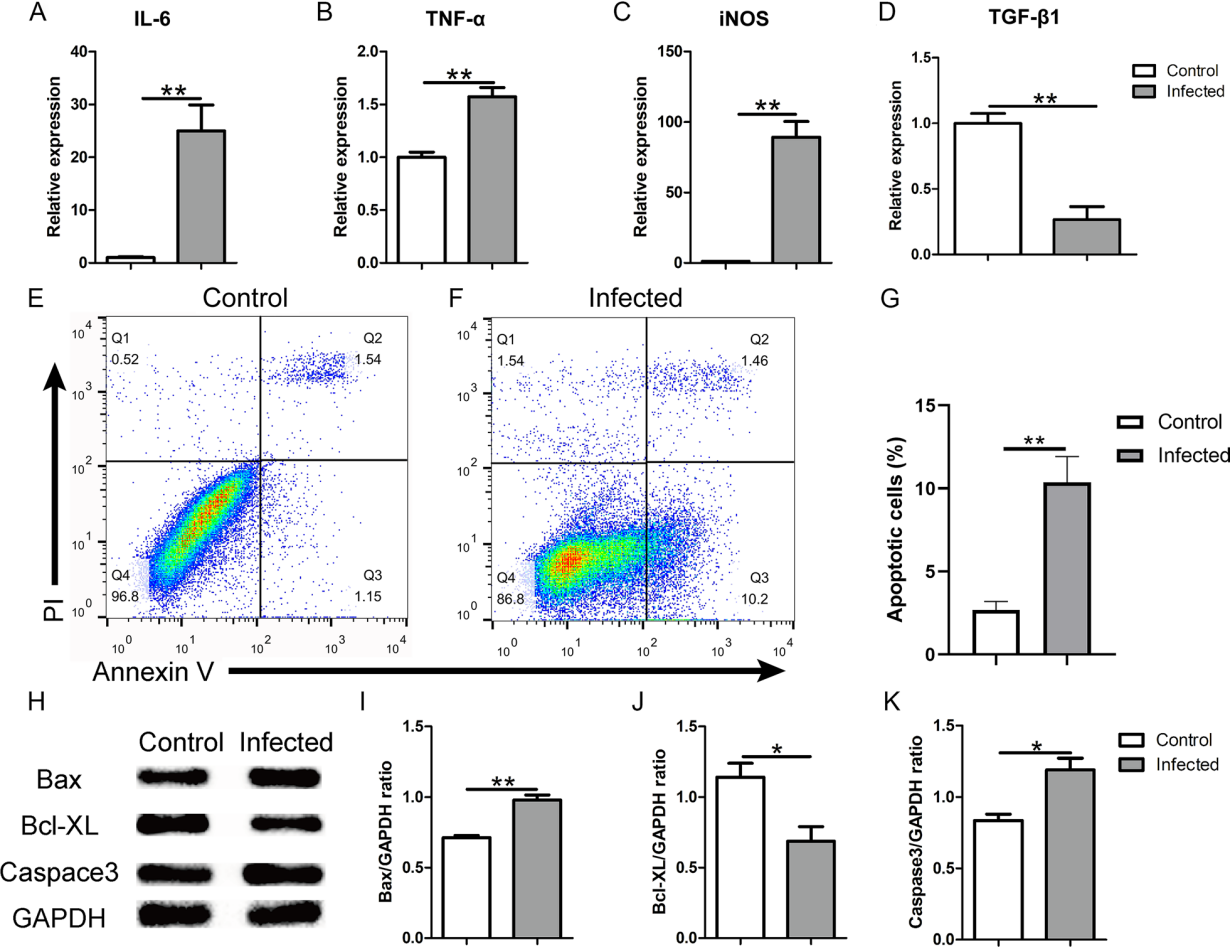
TgCtwh6 tachyzoites indirectly induce HT22 apoptosis by polarizing BV2 into M1. BV2 cells were infected by TgCtwh6 tachyzoites for 24 h; then, RNA of the BV2 was extracted and reverse transcribed to cDNA. The gene expressions of IL-6, TNF-α, iNOS and TGF-β1 were assessed by qRT-PCR and then analyzed semiquantitatively (**A**–**D**). After BV2 cells had been infected with TgCtwh6 tachyzoites for 24 h, the BV2 cells were co-cultured with HT22 for another 24 h in a transwell device. Then, HT22 cells were collected from the lower layer and apoptosis status was assessed using FCM (**E**–**G**). Moreover, the HT22 cells were lysed and proteins were extracted. The expression of apoptosis-related proteins was detected by western blotting and then analyzed semiquantitatively (**H**–**K**). Data were represented as mean ± SEM and were analyzed by two-tailed Student’s *t*-test for comparing the difference between the two groups (*n* = 3 each group). *P < 0. 05, **P < 0.01

As TgCtwh6 tachyzoites can bring about BV2 polarization to M1, we further investigated whether the TgCtwh6 tachyzoite-polarized BV2 would affect HT22 apoptosis. First, we surveyed the inflammatory factors produced by the polarized BV2. Briefly, BV2 cells were infected by TgCtwh6 tachyzoites for 24 h; then, RNA of the BV2 was extracted and reverse transcribed to cDNA. The gene expressions of IL-6, TNF-α, iNOS and TGF-β1 were analyzed by qRT-PCR. The results showed that the gene expressions of pro-inflammatory factor IL-6 (*t*-test: *t*_(4)_ = 8.196, *P* = 0.0012), TNF-α (*t*-test: *t*_(4)_ = 8, *P* = 0.0013) and iNOS (*t*-test: *t*_(4)_ = 7.684, *P* = 0.0015) were noticeably increased; meanwhile, the gene expression of anti-inflammatory factor TGF-β1 (*t*-test: *t*_(4)_ = 7.41, *P* = 0.0018) was notably decreased (Fig. 6A–D). Next, after BV2 cells had been infected by TgCtwh6 tachyzoites for 24 h, they were co-cultured with HT22 in replenished DMEM for another 24 h in a transwell device. Then, HT22 cells were collected from the lower layer of the transwell device and apoptosis status was assessed using FCM. The result showed that, compared with the non-infected BV2 group, the apoptosis rate of HT22 was significantly increased in early and late stages in the infected BV2 group (Fig. 6E–G), indicating that pro-inflammatory factors produced from the polarized BV2 promoted HT22 apoptosis (*t*-test: *t*_(4)_ = 8.123, *P* = 0.0012). Furthermore, the apoptosis-related protein expression in the HT22 was detected by western blotting. The data demonstrated that Bax (*t*-test: *t*_(4)_ = 5.017, *P* = 0.0074) and caspase 3 (*t*-test: *t*_(4)_ = 4.066, *P* = 0.0153) protein expression increased; on the contrary, Bcl-XL (*t*-test: *t*_(4)_ = 3.784, *P* = 0.0194) protein expression decreased in HT22 compared with the control group (Fig. 6H–K). In summary, TgCtwh6 tachyzoite-infected BV2 could be polarized into M1 and produce many pro-inflammatory factors, which caused HT22 apoptosis. This strongly suggested that TgCtwh6 may not only directly, but also indirectly induce hippocampal neuron apoptosis by infecting microglial cells which then generate pro-inflammatory factors in the infected mouse brain.

### TgCtwh6 tachyzoites directly promote HT22 to upregulate BACE1, APP and Aβ expression

**Fig. 7:**
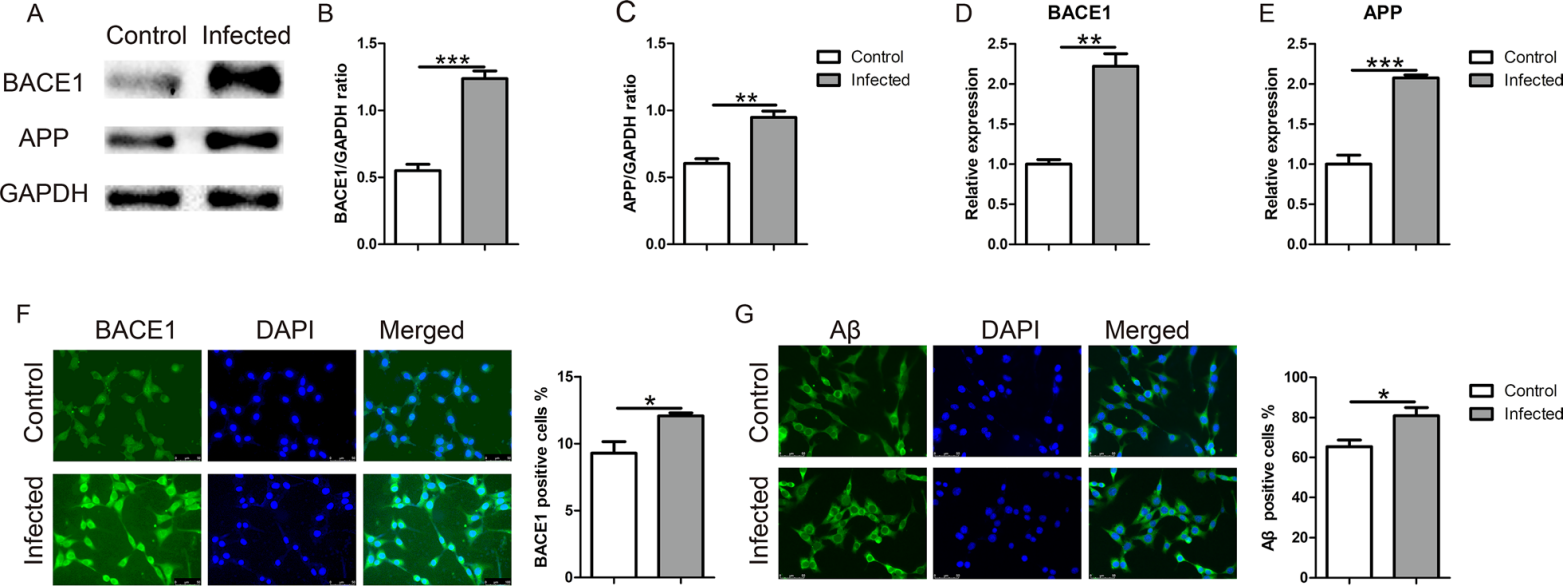
TgCtwh6 tachyzoites upregulate APP, BACE1 and Aβ expression in HT22. After TgCtwh6 tachyzoites had been infected HT22 for 24 h, the HT22 were lysed and proteins were extracted. The expression of BACE1 and APP proteins was measured by western blotting and then analyzed with semiquantitative method (**A**–**C**). Moreover, RNA in the infected HT22 was extracted and reversely transcribed to cDNA. The expressions of BACE1 and APP genes was assessed by qRT-PCR and then semiquantitatively evaluated (**D**, **E**). Furthermore, after the HT22 cells had been fixed and the cytomembranes penetrated by Triton, the BACE1 and Aβ proteins were visualized in situ by immunofluorescence staining and observed under a fluorescence microscope at a magnification of 40 × 100 (scale bars, 50 μm). The percentage of positively stained cells was calculated using morphometric software (**F**–**G**). Data were represented as mean ± SEM and were analyzed by two-tailed Student’s *t*-test for comparing the difference between the two groups (*n* = 3 each group). *P < 0. 05, **P < 0.01, ***P < 0.001

Our in vivo experiments showed that TgCtwh6 infection brought about Aβ deposition in cells and tissue of the hippocampus zone. To clarify whether TgCtwh6 could trigger neurons to produce Aβ protein, we infected HT22 with TgCtwh6 tachyzoites to survey in vitro the mechanism with which the infected neuron generated Aβ protein. First, we investigated whether TgCtwh6 tachyzoites can directly cause Aβ production in HT22. So, we detected BACE1, APP and Aβ protein and gene expression in infected HT22 using western blotting, qRT-PCR and immunofluorescence staining. The results revealed that BACE1 (*t*-test: *t*_(4)_ = 10.13, *P* = 0.0005 for protein; *t*-test: *t*_(4)_ = 7.055, *P* = 0.0021 for gene) and APP (*t*-test: *t*_(4)_ = 7.891, *P* = 0.0014 for protein; *t*-test: *t*_(4)_ = 10, *P* = 0.0006 for gene) protein and gene expression levels were significantly higher in the infected group than in the control group (Fig. 7A–E). Also, percentages of BACE1 (*t*-test: *t*_(4)_ = 3.005, *P* = 0.0398) and Aβ (*t*-test: *t*_(4)_ = 3.343, *P* = 0.0288) protein-positive cell expressions were upregulated in infected HT22 (Fig. 7F, G). These data showed that TgCtwh6 infection could directly lead to the production of Aβ in HT22.

### BV2 cells infected with TgCtwh6 tachyzoites result in APP production in HT22 in co-culture system

**Fig. 8:**
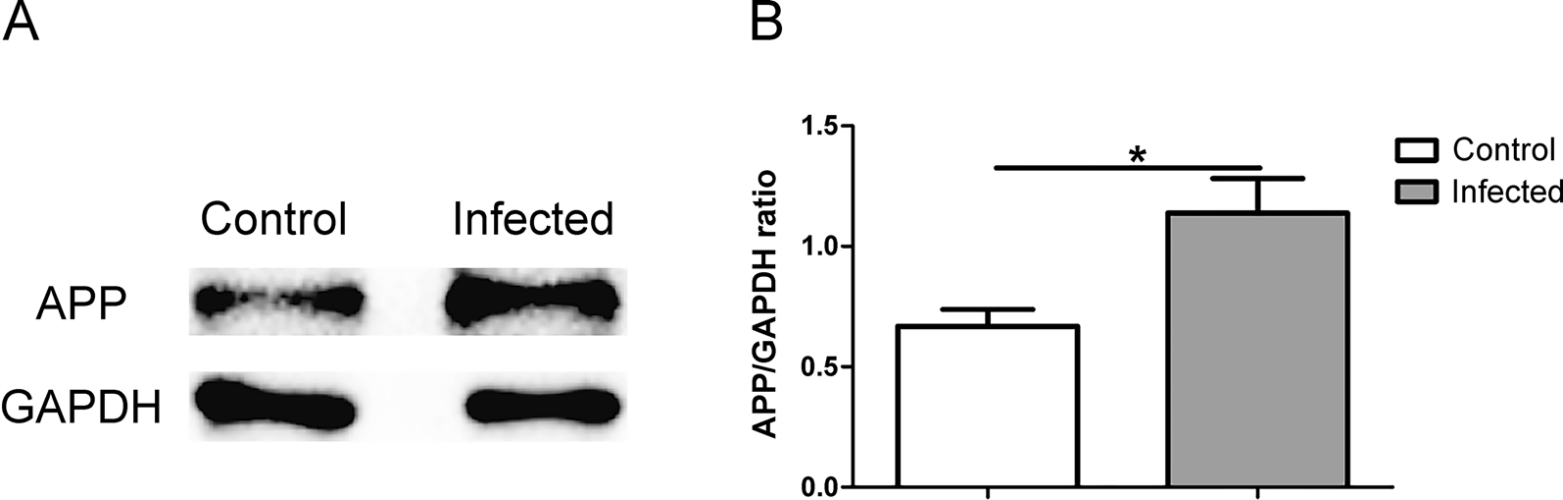
TgCtwh6 tachyzoites indirectly induce APP protein production in HT22 by polarized BV2. BV2 cells infected with TgCtwh6 tachyzoites for 24 h were co-cultured with HT22 for another 24 h in a transwell system. Subsequently, the HT22 cells were collected from the lower layer of the co-culture system and then lysed. The proteins were extracted from the HT22. The expression of APP protein was detected by western blotting and analyzed by semiquantitative method (**A**, **B**). Data were represented as mean ± SEM and were analyzed by two-tailed Student’s *t*-test for comparing the difference between the two groups (*n* = 3 each group). *P < 0.05

Our present experiment showed that TgCtwh6 tachyzoite-polarized BV2 drove HT22 to apoptosis. To clarify whether the TgCtwh6 tachyzoite-polarized BV2 could induce Aβ production in HT22, we investigated the APP protein expression from HT22, which had been co-cultured for 24 h with BV2 infected by TgCtwh6 tachyzoites in a transwell system. Subsequently, we collected HT22 from the lower layer of the co-culture system and analyzed the expression of APP using western blotting. The result showed that APP expression significantly increased (Fig. 8A, B) in HT22 compared with the control group (*t*-test: *t*_(4)_ = 4.128, *P* = 0.0145), suggesting TgCtwh6 may not only directly, but also indirectly induce hippocampal neurons to yield Aβ protein by infecting and polarizing microglial cells in mouse brain.

### TgCtwh6 tachyzoites mediate Aβ production via NF-κB signaling in HT22

**Fig. 9:**
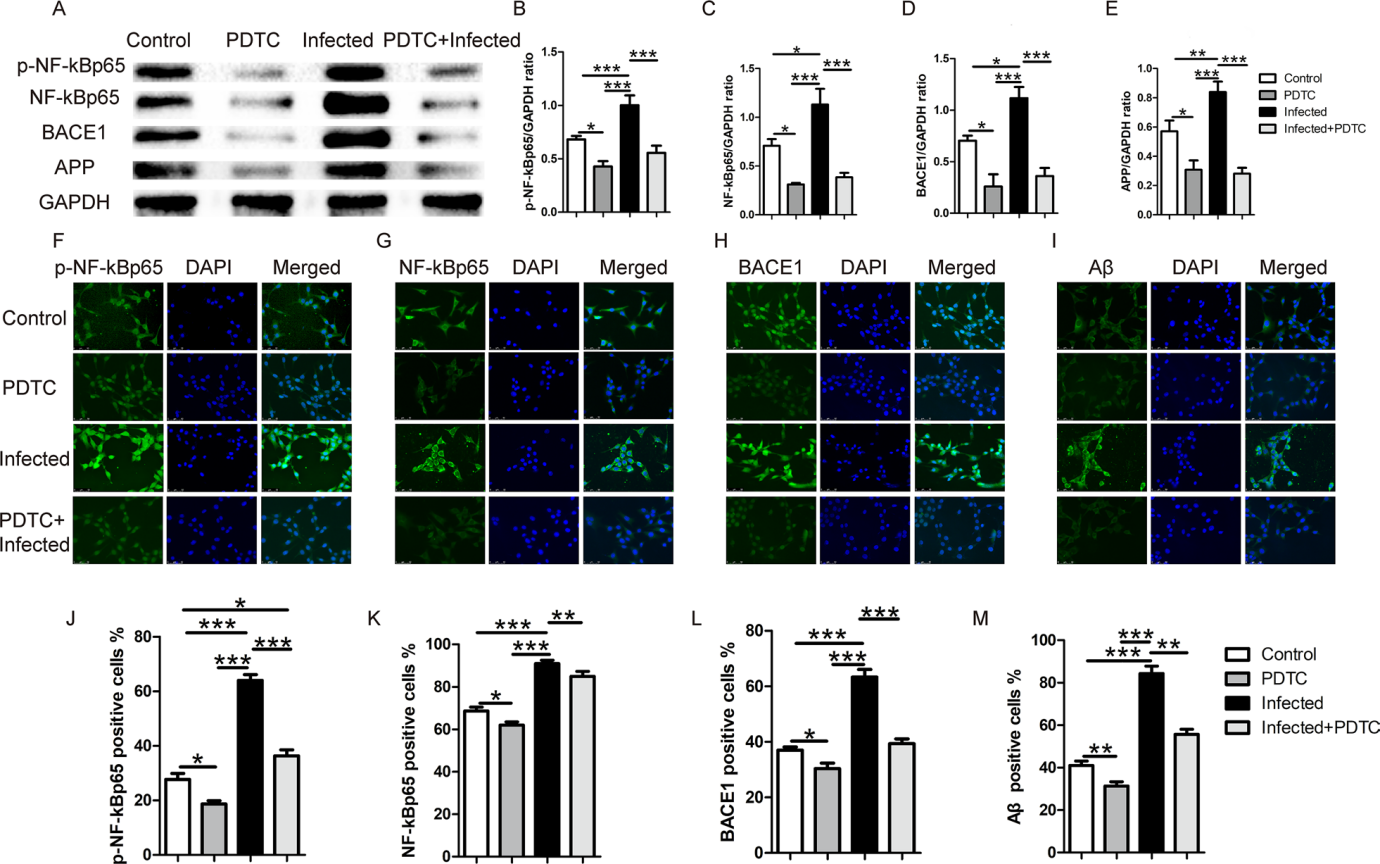
TgCtwh6 tachyzoites induce Aβ production by activating NF-κB signaling pathway in HT22. HT22 cells pre-treated with or without PDTC for 12 h were challenged with or without TgCtwh6 tachyzoites for 24 h. Then, after the HT22 cells had been collected and lysed, the proteins in HT22 were extracted. The expressions of NF-κBp65, *p*-NF-κBp65, APP and BACE1 were detected by western blotting and then semiauantitative analyzed (**A**–**E**). Additionally, after the HT22 cells had been fixed and the cytomembranes penetrated by Triton, the NF-κBp65, *p*-NF-κBp65, BACE1 and Aβ proteins were visualized in situ by immunofluorescence staining and were observed under a fluorescence microscope at a magnification of 40 × 100. The percentage of positively stained cells was calculated using morphometric software (**F**–**M**). Data were represented as mean ± SEM and were analyzed by one-way ANOVA followed by Bonferroni’s post hoc test for multiple comparisons (*n* = 3 each group). *P < 0. 05, **P < 0.01, ***P < 0.001

**Fig. 10:**
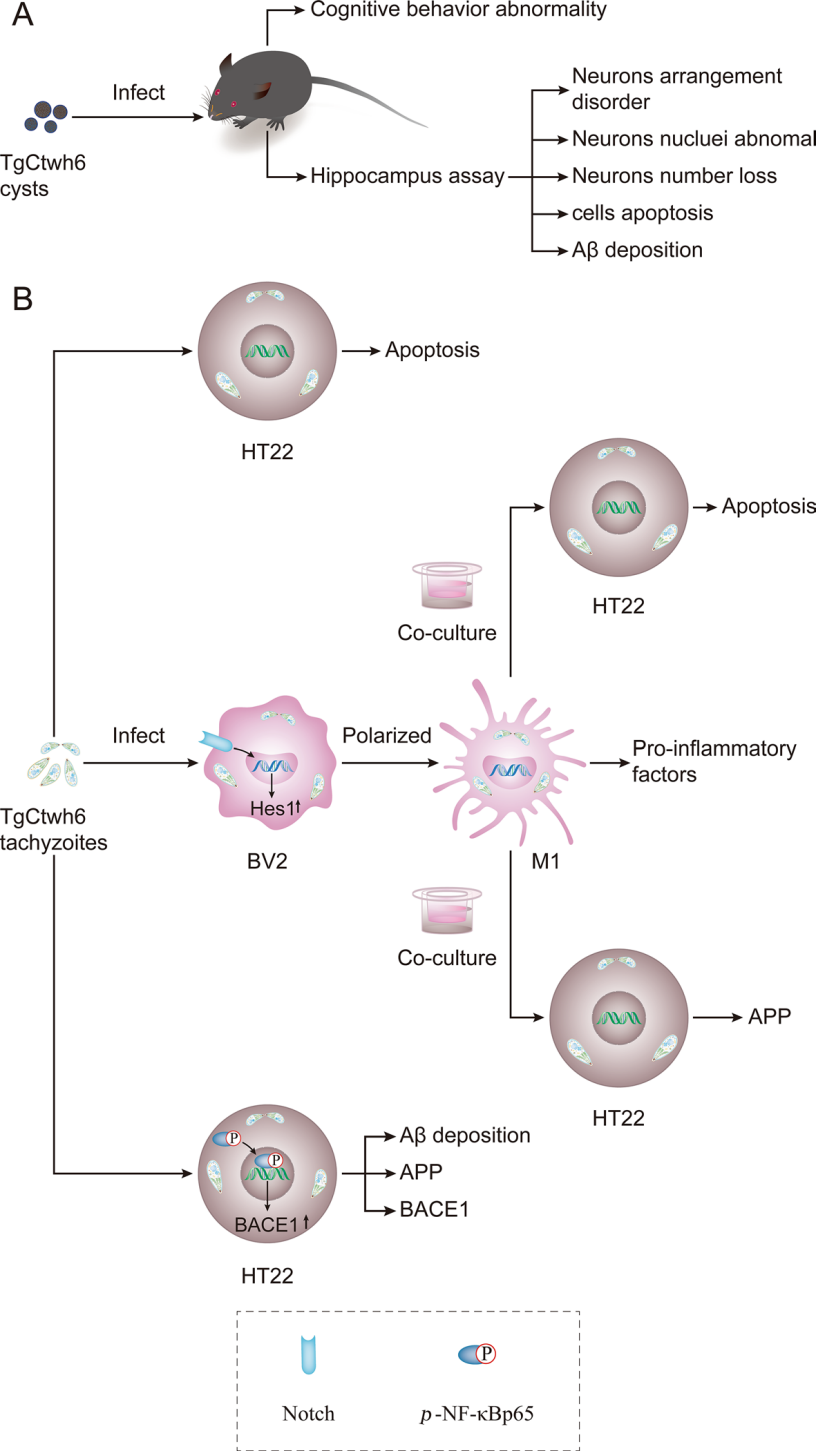
Mechanism with which TgCtwh6 infection induces mouse cognitive behavior disorder at the cellular and molecular level in our experiments. First, in vivo, each C57BL/6 mouse was infected orally with TgCtwh6 cysts. On day 90 post-infection, the infected mice manifested cognitive behavioral abnormalities. The infected mouse hippocampal tissue assay showed that neurons were disorganized in the arrangement, decreased in the number and abnormally stained in nuclei. Moreover, apoptotic cells and Aβ protein increased in the infected mouse hippocampus (**A**). TgCtwh6 tachyzoites led to HT22 apoptosis following HT22 infection for 24 h; meanwhile, TgCtwh6 tachyzoites promoted BACE1, APP and Aβ production by activating NF-κB signaling pathway in HT22. Additionally, after BV2 cells had been infected by TgCtwh6 tachyzoites for 24 h, they could drive polarization to M1 through Notch signaling pathway with production of pro-inflammatory factors, which provoked co-cultured HT22 apoptosis and APP expression (**B**)

To further explore the mechanism by which TgCtwh6 tachyzoites induce Aβ production in HT22, we estimated the effect TgCtwh6 tachyzoites on the production of *p*-NF-κBp65 and NF-κBp65, which are key signaling proteins in the NF-κB signaling pathway in HT22. HT22 cells pre-treated with or without PDTC for 12 h were challenged with or without TgCtwh6 tachyzoites for 24 h. Then, after the HT22 cells had been collected and lysed, the proteins in HT22 were extracted. The expressions of *p*-NF-κBp65, NF-κBp65, BACE1 and APP were detected by western blotting. The data showed that *p*-NF-κBp65 (*F*_(3,8)_ = 53.222, *P* < 0.0001), NF-κBp65 (*F*_(3,8)_ = 29.249, *P* = 0.020), BACE1 (*F*_(3,8)_ = 38.853, *P* = 0.010) and APP (*F*_(3,8)_ = 35.326, *P* = 0.006) proteins increased significantly in the infected HT22 compared with those in non-infected HT22. By contrast, the expression of *p*-NF-κBp65 (*F*_(3,8)_ = 53.222, *P* < 0.0001), NF-κBp65 (*F*_(3,8)_ = 29.249, *P* < 0.0001), BACE1 (*F*_(3,8)_ = 38.853, *P* < 0.0001) and APP (*F*_(3,8)_ = 35.326, *P* < 0.0001) proteins was significantly reduced, respectively, after HT22 cells were pre-treated with PDTC compared with those with infected HT22 without pretreatment (Fig. 9A−E). In addition, the *p*-NF-κBp65, NF-κBp65, BACE1 and Aβ proteins were also detected by immunofluorescence staining. The data revealed that *p*-NF-κBp65 (*F*_(3,8)_ = 85.65, *P* < 0.0001), NF-κBp65 (*F*_(3,8)_ = 77.371, *P* < 0.0001), BACE1 (*F*_(3,8)_ = 82.702, *P* < 0.0001) and Aβ (*F*_(3,8)_ = 89.674, *P* < 0.0001) were markedly highly expressed in infected HT22 compared with those in non-infected HT22. It was noticeable that *p*-NF-κBp65 protein distributed not only in cytoplasm, but also in nucleus of the infected HT22. On the contrary, the expression of *p*-NF-κBp65 (*F*_(3,8)_ = 85.65, *P* < 0.0001), NF-κBp65 (*F*_(3,8)_ = 77.371, *P* = 0.009), BACE1 (*F*_(3,8)_ = 82.702, *P* < 0.0001) and Aβ (*F*_(3,8)_ = 89.674, *P* = 0.001) was evidently decreased after HT22 cells were pre-treated with PDTC compared with those in the infected HT22 without pretreatment (Figs. 9F–M, 10). So, both western blotting and immunofluorescence staining indicated that TgCtwh6 tachyzoites promoted Aβ generation by directly activating NF-κB signaling in HT22.

## Discussion

Chronic *T. gondii* infection has been reported to cause cognitive behavioral abnormalities and is considered a potential cause of many mental illnesses such as AD. At present, although it is known that there is a relationship between *T. gondii* infection and cognitive behavioral abnormality, there is not enough research on the relationship between TgCtwh6 and cognitive behavioral abnormality.

In our studies, we used TgCtwh6 cysts to infect a mouse constructed model of cognitive behavioral abnormalities. Our results indicated that the mice suffering from TgCtwh6 infection showed abnormal appearance and posture. Furthermore, the OFT showed that the infected mice had an obvious corner preference, which is related to increased anxiety. It has been reported that anxiety-like behavior is also common in AD patients [39, 40]; meanwhile, the data from the MWMT revealed that TgCtwh6 infection damaged the mouse spatial memory retention and spatial learning. However, the infected mice had normal mobility, suggesting that *T. gondii* infection hardly affects motor function in mice [41, 42]. After dissecting the brain of mice, cysts were found in the infected group, suggesting that the mice were infected successfully with TgCtwh6. Therefore, in the following experiments, we tried to investigate the mechanism with which TgCtwh6 infection triggers cognitive behavioral abnormalities at the cellular and molecular level.

Our histopathological research showed that the hippocampus neurons were reduced in number, disordered in arrangement and deeply stained in the nucleus in infected mice, suggesting that the changes in morphology and decrease in the number of hippocampal neurons might be related to cognitive behavioral abnormalities. It has been reported that Toxoplasma infection can cause neuron loss in mice [43], and neuron loss, Aβ deposition and neuroinflammation are the vital histopathological features of AD disease [44]. Moreover, neuron apoptosis is a significant cause of neuron loss [45], so we further confirmed in vivo and in vitro whether TgCtwh6 infection can lead to neuronal apoptosis and Aβ deposition in the following experiments.

Our study showed in vivo that Bax and caspase 3 expression increased, while Bcl-XL expression decreased in infected mouse hippocampus tissue, indicating TgCtwh6 infection could cause hippocampal nervous cell (probably including neurons) apoptosis. In addition, the expressions of BACE1, APP and Aβ in mouse hippocampus tissue were significantly upregulated in the infected group, which suggested that Aβ deposition and hippocampal nervous cell apoptosis were implicated in cognitive behavioral abnormalities in mice infected with TgCtwh6. Aβ has a strong neurotoxic effect after abnormal processing and accumulation in the cellular matrix, and it plays an important role in the progression of AD [32].

To verify the mechanism with which TgCtwh6 induces neuron apoptosis and Aβ deposition in neurons, we carried out separate or co-culture experiments in transwell device with HT22 and BV2 in vitro. The results showed that HT22 apoptosis was initiated and Aβ expression was increased in HT22 when the HT22 was infected with TgCtwh6 tachyzoites; moreover, BV2 was activated to M1 with increased expression of IL-6, TNF-α and iNOS when the BV2 was infected with TgCtwh6 tachyzoites. Interestingly, the activated BV2 can induce HT22 apoptosis and APP production in HT22 co-cultured with BV2. In our study, when HT22 was infected with TgCtwh6 tachyzoites, the *p*-NF-κBp65 and NF-κBp65 expressions were upregulated in cytoplasm with *p*-NF-κBp65 also increased in nucleus; meanwhile, BACE1, APP and Aβ production increased, suggesting that Aβ was produced via NF-κB signaling pathway in HT22 infected with TgCtwh6 tachyzoites. Now, more and more studies have affirmed that NF-κB signaling is closely associated with Aβ generation in neurological diseases [46–48]. The *p*-NF-κBp65 interacts with NF-κB binding elements in nucleus to regulate BACE1 at the level of transcription and the BACE1 promoter contains specific *p*-NF-κBp65 binding elements [48].

Additionally, many studies have shown that apoptosis is induced via NF-κB signaling, too. Khalaf et al. reported apoptosis was elicited through NF-κB pathway in cystic fibrosis cells [49]. Shao et al. also showed miR-146a-5p promoted IL-1β-induced chondrocyte apoptosis via the TRAF6-mediated NF-kB pathway [50]. On the contrary, Cheng et al. have demonstrated NF-κB activation after *T. gondii* RH strain infection is involved in the increase of anti-apoptotic gene expression, which plays a pivotal role in the *T. gondii*-mediated blockade of apoptosis [36]. The difference between the two results may be due to the different *T. gondii* gene type and cell strain used in the experiments. Because *T. gondii* type II strain dense granule protein 15 (GRA15_II_), one of the genotype-associated effectors of *T. gondii* II strain, could activate the NF-kB signaling [51], we consider that GRA15_II_ derived from TgCtwh6 might induce HT22 apoptosis and Aβ production through NF-kB signaling. We will further explore this hypothesis in our future work.

Neuroinflammation has been considered as a possible pathological mechanism for cognitive behavioral disorders [1]. Microglia, one of inherent immune cells, is a key mediator of the neuroinflammatory response in brain. Inflammatory response of microglia is an important factor in cognitive behavior abnormalities [1, 52]. In our study, we found that BV2 can be activated into M1 by TgCtwh6 tachyzoites through Notch signaling with the secretion of some pro-inflammatory factors from the polarized BV2, which probably induced HT22 apoptosis and APP production in HT22. Some researchers reported that microglia can be activated by infection (e.g., parasites), trauma and other factors, producing a variety of immune effector molecules that not only mediate chronic inflammation and apoptosis, but also lead to degenerative diseases of the nervous system [53]. The pro-inflammatory cytokines, such as IFN-γ, IL-1β and TNF-α, can attenuate microglia's phagocytic activity and transform microglia into M1 types [54]. Notch signaling is involved in regulating BV2 or microglia activation partly through the cross-talk between TLR4/MyD88/TRAF6/NF-κB pathways in brain damage [55, 56]. Cao et al. showed that, when LPS stimulated BV2 cells, both Notch and NF-κB/p65 protein expression increased significantly, and the expression of Hes-1, TNF-α and IL-1β increased successively. Moreover, they considered Notch signaling can trans-activate NF-κB/p65 by amplifying NF-κB/p65-dependent pro-inflammatory functions in activated BV2 cells [34]. What effector molecule derived from TgCtwh6 tachyzoites can activate Notch signaling, and how does the Notch signaling lead to NF-κB activation, which promotes microglia polarization, neuron apoptosis and Aβ generation in neurons? These are very important and interesting topics on which we will focus in the future.

Some researchers further demonstrated that the pro-inflammatory factors from microglia stimulated by Aβ cause extensive death of apoptotic neurons [21, 56, 57]. Therefore, we supposed that HT22 apoptosis is partly induced by excess Aβ secreted from HT22. Furthermore, Aβ may act as an immune micro-endogenous stimulus in the tissue micro-environment to constantly activate microglia to maintain the M1 pro-inflammatory response.

So, we speculate that neuron apoptosis, Aβ deposition and neuroinflammatory response involved in microglia polarization are the molecular and cellular mechanisms with which TgCtwh6 causes mouse cognitive behavioral abnormalities. We will further verify our speculation in our future work using hippocampal neurons and microglia extracted from infected mice; furthermore, we will use some technologies, such as biochips and in situ hybridization histochemistry, in our future experiments.

## Data Availability

All articles from which data were cited to support the conclusions of this manuscript are listed in the text and the reference.

## Abbreviations

AD: Alzheimer's disease
APP: Amyloid precursor protein
Aβ: Beta-amyloid
BACE1: β-Secretase 1
BV2: Mouse microglial cell line
DAB: 3, 3'-Diaminobenzidine tetrahydrochloride
DAPI: 2-(4-Amidinophenyl)-6-indolecarbamidine dihydrochloride
DMEM: Dulbecco’s modifed Eagle’s medium
FBS: Fetal bovine serum
FCM: Flow cytometry
GRA15_II_: *Toxoplasma gondii* type II strain dense granule protein 15
HE: Hematoxylin and eosin
HFF: Human foreskin fibroblast line
HT22: Mouse hippocampal neuronal cell line
IHC: Immunohistochemistry
MWMT: Morris water maze test
M1: Classically activated macrophage
M2: Alternatively activated macrophage
OFT: Open field test
PDTC: Ammonium pyrrolidinedithiocarbamate
ROP16_I/III_: *Toxoplasma gondii* type I/III strain rhoptry protein 16
RT-qPCR: Quantitative real-time PCR
SPF: Specific pathogen free
TgCtwh3: *Toxoplasma gondii* Chinese 1 genotype Wh3 strain
TgCtwh6: *Toxoplasma gondii* Chinese 1 genotype Wh6 strain
*T. gondii*: *Toxoplasma gondii*
